# Cigarette smoking and risk of rheumatoid arthritis: a dose-response meta-analysis

**DOI:** 10.1186/ar4498

**Published:** 2014-03-05

**Authors:** Daniela Di Giuseppe, Andrea Discacciati, Nicola Orsini, Alicja Wolk

**Affiliations:** 1 Division of Nutritional Epidemiology, Institute of Environmental Medicine, Karolinska Institutet, Nobels vag 13, Stockholm 171 77, Sweden

## Abstract

**Introduction:**

Although previous studies found that cigarette smoking is associated with risk of rheumatoid arthritis (RA), the dose-response relationship remains unclear. This meta-analysis quantitatively summarizes accumulated evidence regarding the association of lifelong exposure to cigarette smoking assessed as pack-years with the risk of RA.

**Methods:**

Relevant studies were identified by a search of MEDLINE and EMBASE from 1966 to October 2013, with no restrictions. Reference lists from retrieved articles were also reviewed. Studies that reported relative risks (RR) or odds ratio (OR) estimates with 95% confidence intervals (CIs) for the association between pack-years of cigarette smoking and rheumatoid arthritis were included in a dose-response random-effects meta-regression analysis.

**Results:**

We included 3 prospective cohorts and 7 case-control studies in the meta-analysis. They included a total of 4,552 RA cases. There was no indication of heterogeneity (*P*_*heterogeneity*_ = 0.32) and publication bias did not affect the results. Compared to never smokers, the risk of developing RA increased by 26% (RR = 1.26, 95% CI 1.14 to 1.39) among those who smoked 1 to 10 pack-years and doubled among those with more than 20 pack-years (RR for 21 to 30 pack years = 1.94, 95% CI 1.65 to 2.27). The risk of RA was not increasing further for higher exposure levels (RR for >40 pack-years = 2.07, 95% CI 1.15 to 3.73). The risk of RA was statistically significantly higher among rheumatoid factor (RF)-positive RA cases (RR = 2.47, 95% CI 2.02 to 3.02) compared to RF-negative (RR = 1.58, 95% CI 1.15 to 2.18) when comparing the highest versus lowest category of pack-years for the individual studies.

**Conclusions:**

Lifelong cigarette smoking was positively associated with the risk of RA even among smokers with a low lifelong exposure. The risk of RA did not further increase with an exposure higher than 20 pack-years.

## Introduction

Cigarette smoking is considered an established risk factor for the development of rheumatoid arthritis (RA), an autoimmune inflammatory disease. This was confirmed by a published meta-analysis on smoking and risk of RA that showed a 40% higher risk among ever smokers compared to never smokers [1]. However, little is known regarding the dose-response relationship between quantity of cigarette smoking and increase in the risk of RA. Previous epidemiological studies showed an increasing RA risk associated with increasing cigarette smoking [2-4], while a recently published study reported that even light smoking was associated with an increased risk of RA [5]. Light smoking as well as heavy smoking could increase the risk of RA due to the triggering of the immune system against citrullinated proteins antigens [6].

The aim of the present study was to quantitatively summarize the accumulated evidence on the relation between lifelong exposure to cigarette smoking and risk of developing rheumatoid arthritis by performing a dose-response meta-analysis. As a measure of lifelong exposure we used pack-years of smoking.

## Methods

We searched MEDLINE and EMBASE databases for relevant studies published from 1966 through October 2013 using the search terms “rheumatoid arthritis” or “RA” combined with “smoke”*,* “smoking”, “cigarette smoking” or “pack-years”, with no restrictions*.* Studies were excluded if they did not meet the selection criteria: use of an observational design (cohort or case-control design), examination of smoking as the main exposure and RA as the main outcome, reporting relative risks or odds ratios of RA for categories of pack-years (obtained by multiplying the number of cigarettes smoked per day by the number of years the person has smoked, divided by 20). In case of multiple publications from the same study, we selected the publication with estimates on the entire study cohort [2,4], instead of the publications focusing only on interactions [7] or anticitrullinated protein antibody (ACPA) positive RA cases [8].

From each study we extracted information regarding publication data (first author’s last name, year of publication and country of the studied population), number of RA cases, cohort size or number of controls, follow-up period for cohorts and study period for case-control studies, exposure specific relative risk or odds ratio estimates with their corresponding 95% confidence intervals (CIs), and variables controlled for in the multivariable model (Table 1). From each study, we extracted the relative risk or odds ratio estimates from the maximally adjusted model to reduce the risk of possible unmeasured confounding. Data were extracted independently by two researchers (DDG and NO).

**Table 1:** Characteristics of studies on rheumatoid arthritis and pack-years

**Authors, year**	**Study population, country, follow-up period/study period**	**Cases/cohort size or controls**	**Range of exposure pack-years**	**Highest category**	RR/OR* Highest vs. lowest category^**§ **^**(95% CI)**	**Controlled variables**	NOQAS*
**Cohort**							
Criswell *et al*., 2002 [20]	Iowa Women’s Health Study, USA, 1986 to 1997	Women 158/31,336	0 to 50^a^	≥40	Total 1.7 (1.0 to 3.0)	Age, body mass index, alcohol use, coffee consumption, marital status, occupation, age at menopause, use of oral contraceptives and hormone replacement therapy.	8
Costenbader *et al*., 2006 [4]	Nurses’ Health Study, USA, 1976 to 2002	Women 680/103,818	0 to 45^a^	>40	Total 1.86 (1.46 to 2.38) RF +^*^ 2.22 (1.63 to 3.02) RF-^*^ 1.43 (0.96 to 2.12)	Age, body mass index, alcohol intake, father’s occupation, age at menarche, menstrual regularity, duration of breastfeeding, postmenopausal hormone use.	8
Di Giuseppe *et al*., 2013 [5]	Swedish mammography cohort, 2003 to 2010	Women 219/34,101	0 to 28^b^	>22	Total 1.82 (1.19 to 2.79)	Age, menopause status, parity, alcohol use, educational level, and body mass index.	8
**Case-control**							
Voigt *et al*., 1994 [16]	Population-based controls, USA, 1986 to 1991	Women 349/1,457	0-27^a^	>20	Total 1.49 (1.06 to 2.10)	Age, body mass index.	7
Hutchinson *et al*., 2001 [24]	Hospital-based controls, UK	Men + Women 239/239	0 to 55^a^	>50	Total 8.41 (2.45 to 28.84)	Matched for age, gender and social class.	7
Olsson *et al*., 2001 [21]	Population-based controls, Sweden, 1980 to 1995	Men 91/203 Women 154/222	0 to 27^a^	≥20	Total men 2.7 (1.2 to 5.8) Total Women 1.8 (0.7 to 4.6) RF + Men 3.4 (1.5 to 8.4) RF + Women 2.5 (0.9 to 6.7)	Age, economic status.	7
Stolt *et al*., 2003 [2]	Population-based controls, Sweden, 1996 to 2000	Men + Women 486/724	0 to 25^a^	≥20	RF + 2.7 (1.8 to 3..9)	Age, residential area and gender.	7
Pedersen *et al*., 2006 [22]	Population-based controls, Denmark, 1988 to 2003	Men 149/291 Women 366/478	0 to 25^a^	>20	Total men 2.00 (1.12 to 3.58) Total Women 2.07 (1.35 to 3.16)	Birth year, year of RA diagnosis, gender.	7
Mikuls *et al*., 2010 [14]	Population-based controls, US (African Americans)	Men + Women 605/255	0 to 15^a^	≥10	Total 2.37 (1.56 to 3.60) RF + 2.51 (1.63 to 3.87) RF- 1.93 (1.11 to 3.35)	Age, gender.	7
Yahya *et al*., 2012 [23]	Population-based controls, Malaysia, 2005 to 2009	Men + Women 1056/1416	0 to 25^a^	≥20	Total 2.3 (1.0 to 5.5)	Matched for age, gender and residential area. Additionally adjusted for formal education and ethnicity.	6

^a^Midpoint in the lowest-highest category ^b^Median of the highest category.

^§^The lowest category corresponded to never smokers in all study except the study of Navarro-Compàn *et al*., that used <20 pack/years as reference level.

*CI, confidence interval; NOQAS, Newcastle-Ottawa Quality Assessment Scale (score from 0 as poor to 9 as excellent); OR, odds ratio; RF+, rheumatoid factor positive; RF-, rheumatoid factor negative; RR, relative risk.

The study quality was assessed using the Newcastle-Ottawa Quality Assessment Scale (NOQAS) for cohort and case-control studies, with which each study was judged based on the selection of the study groups, the comparability of the groups, and the ascertainment of exposure and outcome [9]. The score ranged between 0 (as poor) and 9 (as excellent). The present work follows the recommendations of the PRISMA Statement [10].

### Statistical analysis

We conducted a two-stage dose-response random-effects meta-regression analysis [11,12], and we estimated the dose-response relationship curve by taking into account the covariance among risk estimates for different exposure categories [12]. The possible non-linear relationship between pack-years of smoking and risk of RA was modeled using restricted cubic splines (with three knots). This method requires stating the distribution of cases and non-cases or person-time and the relative risk (RR) or the odds ratio (OR) with its confidence intervals for at least three quantitative exposure categories. For this reason, a study that reported only two exposure categories was excluded from this meta-analysis [13]. For another study that did not provide the distribution of cases and non-cases for each exposure level [14], we used the method proposed by Hamling *et al*. [15] to calculate the pseudo-counts to be able to estimate the covariance of the published ORs. For a study that reported only results stratified by menopausal status [16], we combined the two stratum-specific ORs using inverse variance weighted averages [17]. The midpoint of each exposure category was assigned to each corresponding risk estimate. We assigned the midpoint of the upper open-ended category assuming that they had the same amplitude as the preceding categories, except for one study [5], which reported the median value of the highest category. We assigned a null value to the lowest category, composed in all the studies by never smokers. By testing if the second parameter of the restricted cubic spline model was equal to 0, we observed a limited evidence of a non-linear relationship between pack-years of smoking and RA (*P* = 0.078). However, the restricted cubic spline model resulted in the best fitting model in terms of the Akaike Information Criterion (AIC) when comparing it against a linear model (AIC_splines_ = -70.0; AIC_linear_ = -57.4).

We additionally performed a random-effects meta-analysis comparing the highest versus the lowest category of pack-years within each specific study. For this analysis the approach developed by DerSimonian and Laird was used [17]. We conducted a sensitivity analysis in which one study at a time was removed in order to evaluate the influence of each study on the overall estimate. We also performed analyses stratified by study design (cohort, case-control), gender and rheumatoid factor (RF) type (positive, negative).

In all meta-regression models, statistical heterogeneity between studies was evaluated with the Cochran’s Q-test and the I^2^ statistic [18], which assess the proportion of total variation due to between-study variation. Publication bias was investigated by Egger’s regression asymmetry test [19]. All reported *P*-values are two-sided. All statistical analyses were carried out with Stata, version 12.1 (StataCorp, College Station, TX).

## Results

**Figure 1:**
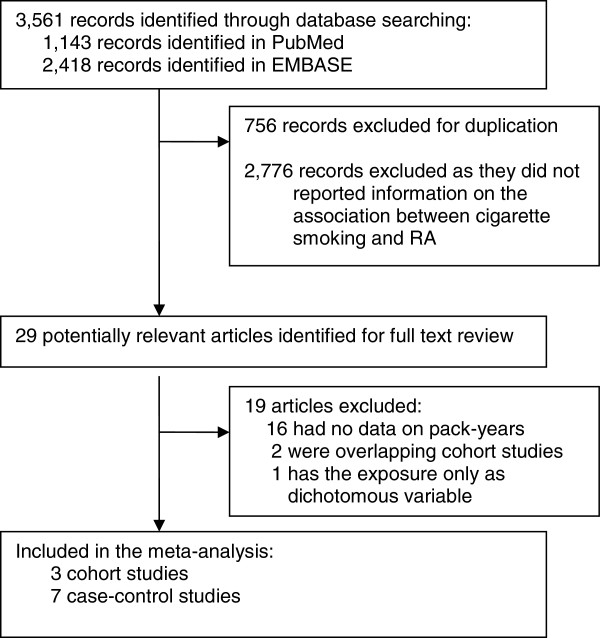
Flow chart of selection of studies for inclusion in the meta-analysis.

Of the 29 studies that reported an estimate of the association between cigarette smoking and rheumatoid arthritis, only 3 cohort studies [4,5,20] and 7 case-control studies [2,14,16,21-24] evaluated the dose-response relationship between pack-years and RA, and were included in this meta-analysis (Figure 1). Of the 10 studies that met the predefined inclusion criteria, 4 were from North America [4,14,16,20], 5 from Europe [2,5,21,22,24], and 1 from Malaysia [23], and they included a total of 181 100 subjects and 4,552 RA cases (Table 1). The median of the highest category of number of pack-years analyzed in each study ranged between 15 and more than 55 pack-years, while the reference group for all studies was never smokers. Four studies provided risk estimates for women only [4,5,16,20] and two studies provided risk estimates for men and women separately [21,22]. Using the NOQAS quality assessment, all 10 studies were assessed to have moderate quality (Table 1). All studies included in this meta-analysis showed a significant dose-response increased risk, except one [21], which reported a non-significant positive association between RA and cigarette smoking among women.

**Figure 2:**
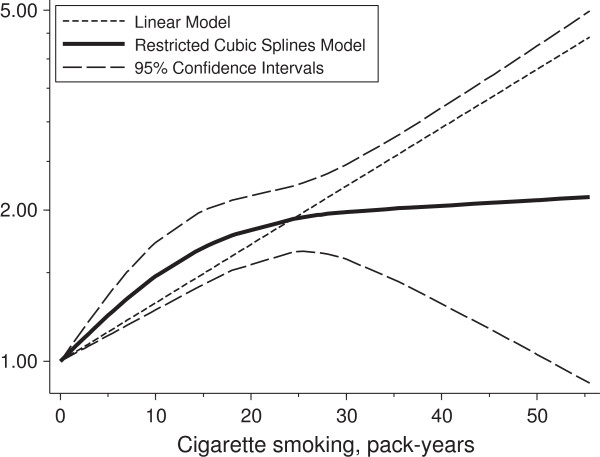
**Non-linear dose-response relationship between pack-years of cigarettes smoking and relative risk of RA.** Relative risk (solid line) and 95% confidence interval (long dashed lines) from the restricted cubic splines model. The short dashed line represents the RR from the linear model. Estimates reported in the table are based on median value of each category.

In a dose-response analysis, we modeled the relationship between pack-years of cigarette smoking and RA risk using a restricted cubic splines model. The non-linear dose-response trend (Figure 2) showed a statistically significant increased risk of developing RA with increasing number of pack-years smoked up to 20 pack-years, and then the relative risk stabilized approximately at the value of 2. The risk of RA was 26% higher (RR = 1.26, 95% CI 1.14 to 1.39) for those who smoked 1 to 10 pack-years of cigarette (median value 5.5) compared to never smokers, while the risk was two-fold for those smoking 21 to 30 pack-years (RR = 1.94, 95% CI 1.65 to 2.27) and was similar even for higher levels of cigarette smoking (RR for >40 pack-years = 2.07, 95% CI 1.15 to 3.73) (Table 2).

**Table 2:** Relative risks (RRs) and 95% confidence intervals (95% CIs) for categories of pack-years of smoking

**Pack-years**	**RR (95% CI)**
Never smokers	1.00
1 to 10	1.26 (1.14 to 1.39)
11 to 20	1.70 (1.44 to 2.01)
21 to 30	1.94 (1.65 to 2.27)
31 to 40	2.02 (1.44 to 2.82)
>40	2.07 (1.15 to 3.73)

The comparison of the highest vs. the lowest categories of pack-years of smoking from the individual studies was in line with the results from the dose-response analysis showing a two-fold increase of RA risk (overall RR = 2.02, 95% CI 1.75 to 2.33). A sensitivity analysis excluding one study at a time showed that the overall RRs ranged between 1.92 and 2.10. Q test for heterogeneity of the results between specific studies was not statistically significant (Q = 12.61, *P*_*heterogeneity*_ = 0.32). There was no evidence of publication bias (*P*-value from the Egger’s regression asymmetry test was 0.10).

**Figure 3:**
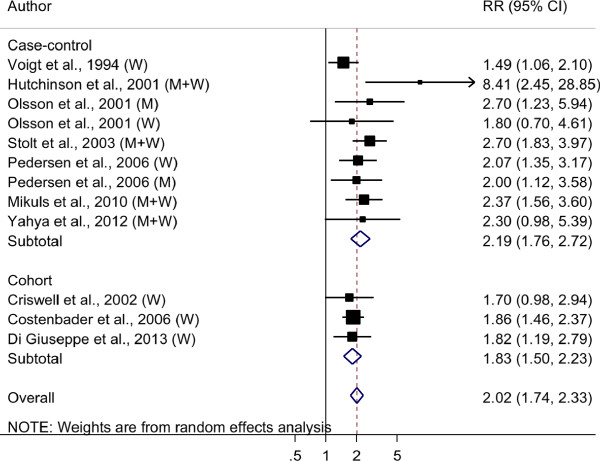
**Forest plot of relative risk estimates for rheumatoid arthritis risk associated with pack-years of cigarette smoking (highest vs. lowest category).** CI indicates confidence intervals; RR indicates relative risk. The size of each square is proportional to the study’s weight (inverse variance). *Test for heterogeneity: cohort studies, *P* = 0.96, I^2^ = 0.0%; case-control studies, *P* = 0.19, I^2^ = 28.7%, overall studies, *P* = 0.32, I^2^ = 12.7%.

We conducted stratified analyses by study design, gender and rheumatoid factor type. The RR comparing the highest vs. the lowest category of pack-years of smoking was 1.83 (95% CI 1.50 to 2.23; Q = 0.09, *P*_*heterogeneity*_ = 0.96) among cohort studies and 2.19 (95% CI 1.76 to 2.72; Q = 11.22, *P*_*heterogeneity*_ = 0.19) among case-control studies (Figure 3). The RR estimate was 1.78 (95% CI 1.52 to 2.08; Q = 1.69, *P*_*heterogeneity*_ = 0.89) among women and 2.22 (95% CI 1.39 to 3.55; Q = 0.36, *P*_*heterogeneity*_ = 0.55) among men. Four studies reported estimates for RF-positive RA [2,4,14,21] and only two for RF-negative RA [4,14]. Among RF-positive cases the RR comparing the highest vs. the lowest category of pack-years of smoking was 2.47 (95% CI 2.02 to 3.02; *P*_*heterogeneity*_ = 0.88), while it was 1.58 (95% CI 1.15 to 2.18, *P*_*heterogeneity*_ = 0.39) among RF-negative cases. These estimates were statistically significantly different (*P*-value 0.022).

## Discussion

The findings from this dose-response meta-analysis showed some evidence of a non-linear association between lifelong cigarette smoking and RA. The association was statistically significant even among those smoking less than 10 pack-years. For more than 20 pack-years the risk of RA was two-fold compared to never smokers and it stabilized with no further increase for higher levels. The risk among RF-positive RA cases was statistically significantly higher than the risk among those RF-negative.

The dose-response meta-analysis was based on a limited number of studies. Only 10 studies satisfied the inclusion criteria, of which only 3 were prospective cohort studies. In the analyses we did not observe evidence of heterogeneity. Publication bias did not affect the findings of this meta-analysis. Results from this meta-analysis are in line with the results from the previous meta-analysis [1], showing an increased RA risk associated with cigarette smoking. However, this meta-analysis of the dose-response relationship between cigarette smoking and RA can allow a better understanding of the association.

Among the limitations of the present study, we have to consider the limited number of studies addressing the dose-response association between lifelong exposure to cigarette smoking and RA. Moreover, a meta-analysis is influenced by the quality of the summarized studies. The quality scale used in this manuscript indicates a moderate quality for all the studies. However, this quality scale does not take into account the differences between case-control and prospective cohort design, such as different biases (for example, case-control studies may be affected by recall bias, while prospective cohort are not). Moreover, the pooled estimate from this meta-analysis could have been affected by a potential residual confounding from each study. In particular, the numbers of confounders taken into account by the case-control studies included in this meta-analysis were in general limited. This could explain the difference between the pooled OR from case-control studies and the pooled RR from prospective cohort studies. Moreover, this difference could also be explained by the different study populations: prospective cohort studies analyzed only women while all case-control studies except one [16] included both men and women.

The biological effect of smoking in the development of rheumatoid arthritis is still unclear. However, it is known that certain cigarette smoke components, such as nicotine, hydrocarbons and carbon monoxide, may enhance immune reactions, and it has been found that in certain inbred strains of rodents these components cause arthritis without contributions from other agents [25-27]. Moreover, it has been hypothesized that smoking interacts with *HLA-DR SE* genes in triggering and persuading immunity against citrullinated proteins, and that this immunity is specific for RA [6,28]. The observed non-linear shape of the dose-response association in this meta-analysis is compatible with this triggering mechanism. RF-positive RA is more likely to be associated with the HLA-DR4 shared epitope [29] than RF-negative RA. This can explain the difference in risk between RF-positive and RF-negative RA cases observed in the meta-analysis.

## Conclusion

Our study showed some evidence of a non-linear dose-response relationship between lifelong smoking and risk of RA. The risk increased at a relatively low level of lifelong exposure to smoking (≤10 pack/years) and stabilized to approximately a double risk for a smoking exposure higher than 20 pack-years.

## Abbreviations

ACPA: anticitrullinated protein antibody; AIC: Akaike Information Criterion; CI: Confidence interval; NOQAS: Newcastle-Ottawa Quality Assessment Scale; OR: Odds ratio; RA: Rheumatoid arthritis; RF: Rheumatoid factor; RR: Relative risk.

## Competing interest

The authors declare that they have no competing interests.

## Authors' contributions

DDG, AD, NO and AW participated in the study design and in writing the manuscript. DDG, AD and NO participated in the data collection. DDG analyzed the data and wrote the manuscript under the supervision of AW. DDG, AD, NO and AW interpreted the data and critically reviewed the paper. All authors read and approved the final manuscript.
